# Simazine, a triazine herbicide, disrupts swine granulosa cell functions

**DOI:** 10.21451/1984-3143-2017-AR960

**Published:** 2018-08-16

**Authors:** Francesca Grasselli, Simona Bussolati, Roberto Ramoni, Stefano Grolli, Giuseppina Basini

**Affiliations:** Dipartimento di Scienze Medico-Veterinarie, , , .

**Keywords:** simazine, endocrine disruptor, swine granulosa cells, ovarian function, angiogenesis

## Abstract

The triazine herbicide simazine is a pesticide commonly detected in surface and ground waters, although banned in most European countries since 2004. Concerns for humans and animal health result from its potential endocrine disrupting action, that can lead to reproductive disorders. The present *in vitro* study was undertaken to study simazine effects on swine granulosa cell function, namely cell viability, proliferation, steroidogenesis and NO production. Moreover, the ability of this substance to interfere with the angiogenetic process, a crucial event in reproductive function, was taken into account. Our data document that simazine treatment, at 0.1 or 10 μM concentration levels, stimulates granulosa cell proliferation and viability and impairs steroidogenesis, increasing in particular progesterone production. In addition, the *in vitro* angiogenesis bioassay revealed a significant simazine stimulatory effect on immortalized porcine Aortic Endothelial Cell proliferation. Collectively, these results show that simazine can display disruptive effects on ovarian cell functional parameters, possibly resulting in reproductive dysfunction. This hypothesis is also supported by the observed pro-angiogenetic properties of this herbicide, as already suggested for different endocrine disruptors.

## Introduction

Simazine belongs to the triazine family, a group of chemicals which are widely employed as broad-spectrum herbicides due to their inhibition of electron transfer in photosynthesis ( Qian *et al*., 2014 ). Owing to their effectiveness, these herbicides have been heavily used in the United States, Europe and Australia for more than 50 years ( Breckenridge *et al*., 2016 ). Compared to other herbicides, triazines are more soluble in water and they can leach from soils to surface and ground waters; thus, contamination of drinking water can raise concerns both for human and animal health.

Atrazine and simazine have gained attention in the field of water policy since 2001 (European Parliament and Council of European Union, 2001). In most European countries their registrations were canceled ( Commission of the European Communities, 2004a and b ) due to their persistence in the environment and mobility in soil and ground water ( Thurman *et al*., 1992 ). In particular, simazine represents the second most commonly detected pesticide in surface and ground waters in different regions worldwide ( Sai *et al*., 2015 ).

Although in Italy it has been banned from use from 2005, the recent 2016 Report of Istituto Superiore per la Protezione e la Ricerca Ambientale (ISPRA) still reported the presence of simazine in both surface and ground water (ISPRA Rapporto nazionale pesticidi nelle acque), especially in the river Po valley. In the U.S., where triazine herbicides are still used, atrazine and simazine have been reported above the maximum admitted contaminant levels in groundwater ( USEPA, 2006 ).

Due to structural similarity to atrazine, simazine and other major chlorometabolites have been classified as a Common Mechanism Group with disrupting effects on hypothalamic-pituitary-gonadal axis ( USEPA, 2006 ). However, to date only few studies have focused on simazine potential adverse health effects and further examinations are therefore required.

Concerns have been raised about the endocrine disrupting effects of triazines and their chlorometabolites, whose adverse effects have been documented in different species. In particular, both atrazine and simazine affects the hypothalamic-pituitary-gonadal axis inducing variations in ovulatory cycles and constant estrus in female rats ( Cooper *et al*., 2000 ). Moreover, chlorotriazines have been involved in the increased risk of mammary tumors resulting from continuous estrogen stimulation of mammary gland ( Stevens *et al*., 1994 ; Fuhrman *et al*., 2012 ). In the mouse, Park *et al*. (2014) recently documented impaired development and growth in offsprings resulting from maternal exposure to low levels of simazine during gestation. Increased apoptosis and decreased proliferation in mouse ovaries have also been shown ( Park *et al*., 2014 ), as well as a significant delay of the onset of puberty in rats after simazine treatment ( Zorrilla *et al*., 2010 ).

Even if *in vitro* studies have highlighted time- and dose-dependent effects on steroidogenesis in murine Leydig cell lines ( Forgacs *et al*., 2013 ) and a disruption on relaxin signaling and nitric oxide production in human granulosa cells ( Park *et al*., 2016 ), a comprehensive understanding of simazine effects on ovarian physiology is lacking. Granulosa cells represent a valuable model to investigate ovarian function: they are essential for ovarian follicle growth, steroidogenesis, oocyte survival and nourishment. Moreover, they are an interesting example of angiogenesis; they are the unique site of physiological neovascularization in the adult that occurs cyclically in the ovary, driving the normal development and growth of ovarian follicles ( Basini *et al*., 2008 ).

This *in vitro* study was set up to verify if simazine can display disrupting effects on swine granulosa cell function, namely cell viability, proliferation, steroidogenesis and NO production.

By means of a previously validated three-dimensional *in vitro* angiogenesis assay ( Basini *et al*., 2016 ), we also verified the ability this substance to interfere with the angiogenetic process, which represents a crucial event involved in ovarian follicles physiological development.

## Materials and Methods

All reagents were obtained from Sigma (St. Louis, MO, USA) unless otherwise specified.

### Granulosa cell collection

Swine ovaries were collected at a local slaughterhouse from Large White cross-bred gilts, parity = 0. The stage of the cycle was unknown. Follicles were classified on a dimension-based fashion ( Basini *et al* ., 2008 ). The ovaries were placed into cold PBS (4°C) supplemented with penicillin (500 IU/mL), streptomycin (500 μg/mL) and amphotericin B (3.75 μg/mL), maintained in a freezer bag and transported to the laboratory within 1 h. After a series of washings with PBS and ethanol 70%, granulosa cells were aseptically harvested by aspiration of large follicles (>5 mm) with a 26-gauge needle and released in medium containing heparin (50 IU/mL), centrifuged for pelleting and then suspended in 0.9 percent (W/V) prewarmed ammonium chloride at 37°C for 1 min to remove red blood cells. Cell number and viability were estimated using a haemocytometer under a phase contrast microscope after vital staining with trypan blue 0.4% of an aliquot of the cell suspension. Cells were seeded in culture medium (CM) represented by DMEM/Ham's F12 (Gibco, Grand Island, NY, USA) supplemented with sodium bicarbonate (2.2 mg/mL), bovine serum albumin (BSA 0.1%), penicillin (100 IU/mL), streptomycin (100 μg/mL), amphotericin B (2.5 μg/mL), selenium (5 ng/mL) and transferrin (5 μg/mL). Once seeded, cells were incubated in the presence or absence of simazine (0.1 or 10 μM). The tested concentrations were chosen on the basis of the expected ovarian concentrations after simazine exposure ( Forgacs *et al*., 2013 ). Ethanol was used as the carrier solvent and its final concentration was less than 0.1% v/v, a level that has no effects on the examined parameters. Cells were then maintained for 48 h at 37°C under humidified atmosphere (5% CO_2_). This procedure was identical for all experiments performed in this study.

### Granulosa cell viability

Intracellular ATP level was measured using a luminescence ATP detection assay (ATPlite Perkin Elmer Inc., Waltham, MA, USA) according to the supplier's instruction. ATP is a marker for cell viability because it is present in all metabolically active cells and its concentration declines very rapidly when the cells undergo either necrosis or apoptosis. Briefly, each lyophilized substrate solution vial was reconstituted with 5 mL substrate buffer and shaken gently until homogenous, then aliquoted and stored at −20°C. Similarly, 1170 μL of sterile distilled water was added to a lyophilized ATP standard vial to get a final10 mM concentration level. Then, six different ATP dilutions (10^−3^ –10^−8^ M concentration range) were prepared in distilled water and stored at −20°C. Granulosa cells (2×10^5^ cells per 200 μL CM/well) were grown in 96-well microplates and incubated for 48 h in the presence or absence of simazine (0.1 or 10 μM). For the ATP assay, all reagents (substrate solution, ATP dilution series, and mammalian cell lysis solution) were equilibrated at room temperature. For the ATP standard solutions, 100 μL culture media/well were placed into a 96-well plate. Then 50 μL of mammalian cell lysis solution which stabilize the ATP were added; 10 μL from each ATP dilution series were added to the well containing only culture media and the microplate was shaken for 5min on an orbital shaker at 700 rpm at room temperature. Finally, 50 μL of the substrate solution was added, and the microplate was shaken again for 5 min at 700 rpm. The plate was covered with an adhesive seal, dark-adapted for 10 min and luminescence was measured using a luminescence microplate reader (Multilabel Counter Victor, Perkin Elmer, Boston, USA). The ATP standard curve was obtained by plotting the luminescence signal of the different ATP dilutions versus the ATP concentrations. The signal for unknown sample was determined obtained by linear regression analysis.

### Granulosa cell proliferation

10^4^ cell/well were seeded in 96-well plates in 200 μL CM and treated with the simazine concentrations as above indicated. Cell proliferation was evaluated by DELFIA 5-bromo-2’-deoxyuridine (BrdU) incorporation assay test (Roche, Mannheim, Germany). Briefly, after 44 h of incubation in the presence or absence of treatments, 20μL BrdU were added to each well, then culture media were removed and a DNA denaturating solution was added in order to improve the accessibility of the incorporated BrdU for antibody detection. Thereafter, 100μL anti-BrdU antibody solution were added to each well. After a 1.5 h incubation at room temperature, 200 µl of DELFIA inducer were added and fluorescence emission were recorded by means of Victor. To quantify viable cell number, the fluorescence emission level of each sample was related to a standard curve prepared by culturing, in quintuplicate, granulosa cells at different plating densities (from 10^3^ to 10^5^ /200 μL) for 48 h. The curve was repeated in four different experiments. The relationship between cell number and absorbance was linear (r = 0.92). Cell number/well was estimated from the resulting linear regression equation. The assay detection limit was 10^3^ cell/well and the variation coefficient was less than 5%.

### Steroid production

10^4^ cells/well were seeded in 96-well plates in 200 μL CM. Since androgens are needed for estradiol synthesis by granulosa cells, CM was supplemented with androstenedione (28 ng/mL) ( Basini and Tamanini, 2000 ) in order to support biosynthetic pathway. Cells were incubated for 48 h in the presence or absence of simazine (0.1 or 10 μM). Culture media were then collected, frozen and stored at −20°C until progesterone (P4) and 17β estradiol (E2) determination by validated RIAs ( Grasselli *et al* ., 1993 ).

P4 assay sensitivity and ED50 were 0.24 and 1 nmol/L, respectively; E2 assay sensitivity and ED50 were 0.05 and 0.2 nmol/L. The intra- and inter-assay coefficients of variation were less than 12% for both assays.

### NO production

10^5^ cells/200 μl CM were seeded in 96-well plates and incubated for 48 h in the presence or absence of simazine (0.1 or 10 μM). NO was assessed by measuring nitrite levels in culture media by the microplate method based on the formation of chromophore after reaction with Griess reagent, which was prepared fresh by mixing equal volumes of stock A (1.0% W/V sulfanilamide, 5.0% W/V phosphoric acid in water) and stock B (0.1% W/V N-[naphthyl] ethylenediamine dihydrochloride in water) ( Dong and Yallampalli, 1996 ) solutions. After incubation with Greiss reagent the absorbance was determined with the Victor Reader using a 540 nm against 620 nm filter. A calibration curve ranging from 0.39 to 25 μΜ was prepared by diluting sodium nitrite in culture medium.

### The three-dimensional in vitro angiogenesis assay

The immortalized porcine aortic endothelial cell line (AOC) used in the experiments was kindly provided by Prof. Jose Yélamos (Hospital Universitario Virgen de la Arrixaca, El Palmar, 30120 Murcia, Spain). The cells were used at 13° passage, grown in Medium 199 (containing Earle’s salts and L-glutamine) supplemented with sodium bicarbonate (2.2 mg / mL), penicillin (100 IU / mL), streptomycin (100 mg / mL), amphotericin B (2.5 mg / mL) and 20% W/V FBS (Fetal Bovine Serum) (GIBCOTM, Invitrogen Corporation, UK) and incubated at 37°C in a humidified atmosphere (5% CO_2_).

### Fibrin gel angiogenesis assay

The model used to study vascular development ( Grasselli *et al*., 2003 ) was prepared using AOC grown on dextran beads coated by denatured collagen from porcine skin (citodex-3 microcarrier beads, MC), included in a gelatinous matrix of fibrin. The first stage of gel preparation involves the adhesion of cells to MC; to this, 1.25 mg of MC were incubated for 3 hours at 37°C with 1.5 mL of sterile PBS in order to achieve optimum hydration. After a first washing with sterile PBS and a second one with Medium 199, the MC were put into a flask with 5 mL culture medium, and AOC (5 ×10^5^) were added. The flask was then incubated overnight at 37°C, to allow cell adhesion on MC surface. Then, fibrin gels were prepared in 12 well plates, adding to each well, in the following order: 873 µl of a fibrinogen solution (1 mg/mL PBS), 20 µL of suspension of AOC coated MC, 128 µL of thrombin (5 U/mL). Fibrin gel polymerization was obtained by incubation for 30 min at 37°C, followed by an 1 hour balancing step with 2 mL of Medium 199. Thereafter, the medium was removed with an insulin syringe and replaced by Medium 199 + 20% FBS, containing simazine (0.1 or 10 µM). After 48 hours, media and treatments were renewed and the plates were incubated for additional 48 h. Endothelial buds proliferation starting from MC was quantified through the software for image processing, Scion Image Beta 4:02 (Scion Corporation, MA, USA, http://rsb.info.nih.gov/nih-image ). After 48 and 96 hours of incubation five photographic images of each gel were acquired, each containing two or three MC; images were then converted to grayscale, reduced by 50% (Paintbrush Software, MS Office) and saved as 24-bit Bitmap, compatible with the Scion software. The measurements, in pixels, were made by drawing the perimeter of the area occupied by the AOC. The validity of this method of quantification of AOC proliferation was confirmed by evaluating the correlation between the area covered by the AOC in fibrin gel and the number of cells actually present in the same area ( Basini *et al*. 2008 ).

### Statistical analysis

Each experiment was repeated at least 6 times with 6 replicates for each treatment. Experimental data are presented as mean ± SEM; statistical differences were calculated with analysis of variance using the Statgraphics package (STSC Inc., version 5.1, Rockville, MD, USA). When significant differences were found, means were compared by Scheffè’s F test. P values < 0.05 were considered to be statistically significant.

## Results

### Effects on granulosa cell viability and proliferation

**Figure 1:**
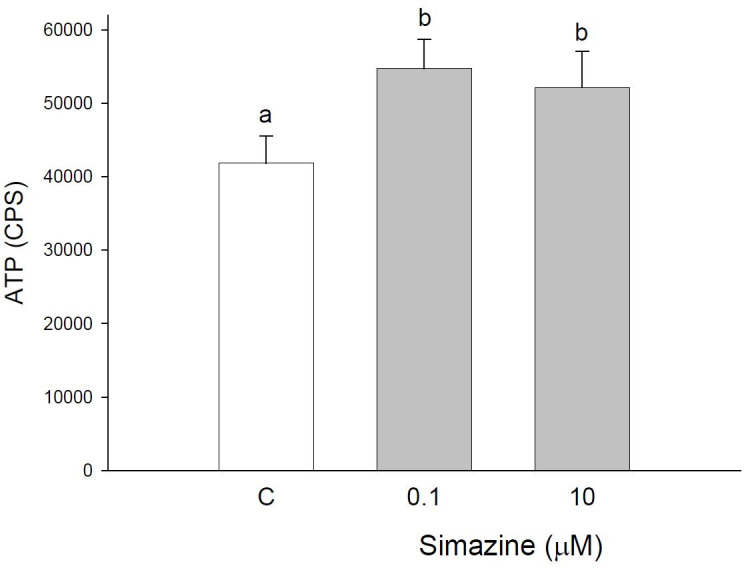
Effect of the 48 h treatment with simazine (0.1 or 10 µM) on ATP production by granulosa cells collected from large (>5 mm) follicles. Data represent the mean ± SEM of six replicates/treatment repeated in six different experiments. Different letters indicate a significant difference (P < 0.05) among treatments as calculated by ANOVA and Scheffè’ F test.

ATP detection assay has shown that simazine is effective (P < 0.05) in stimulating granulosa cell viability ( Figure 1 ). The two concentrations tested displayed similar effects ( Figure 1 )

**Figure 2:**
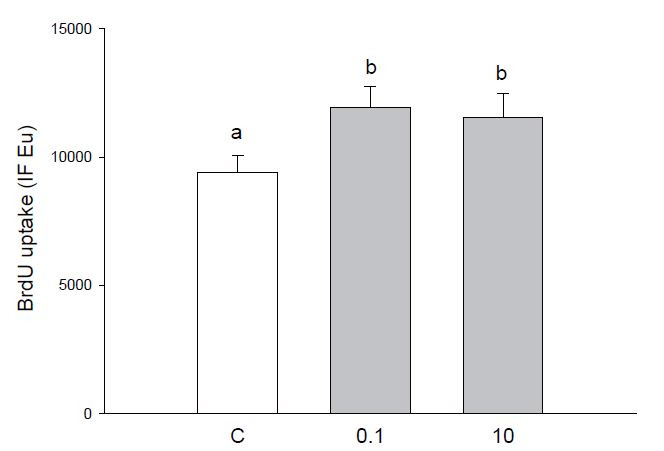
Effect of the 48 h treatment with simazine (0.1 or 10 µM) on BrdU uptake in granulosa cells collected from large (>5 mm) follicles. Data represent the mean ± SEM of six replicates/treatment repeated in six different experiments. Different letters indicate a significant difference (P < 0.05) among treatments as calculated by ANOVA and Scheffè’ F test.

Granulosa cell proliferation was significantly (P < 0.05) increased by simazine, without remarkable differences between the two concentrations ( Figure 2 ).

### Effects on granulosa cell steroidogenesis

**Figure 3:**
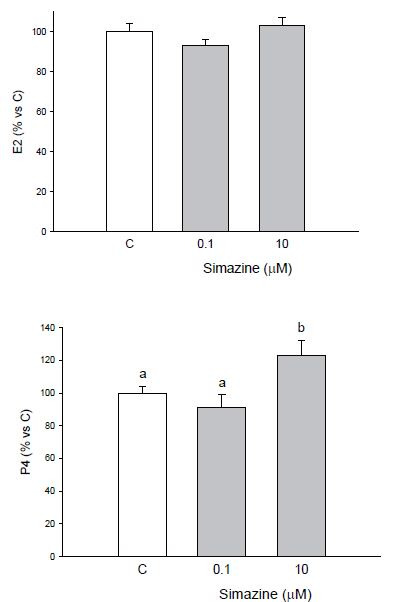
Effect of the 48 h treatment with simazine (0.1 or 10 µM) on E2 (A) or P4 (B) output by granulosa cells collected from large (>5 mm) follicles. Data represent the mean ± SEM of six replicates/treatment repeated in six different experiments. Different letters indicate a significant difference (P < 0.05) among treatments as calculated by ANOVA and Scheffè’ F test.

As for steroid production, simazine did not modify estradiol levels as compared to controls ( Figure 3a ), while progesterone production was stimulated only in granulosa cells incubated with 10 μM simazine (P < 0.05; Figure 3b ).

### Effects on granulosa cell NO production

Neither 0.1 nor 10 μM simazine displayed modulatory effects on NO production by granulosa cells (data not shown).

### Effects on AOC growth

**Figure 4:**
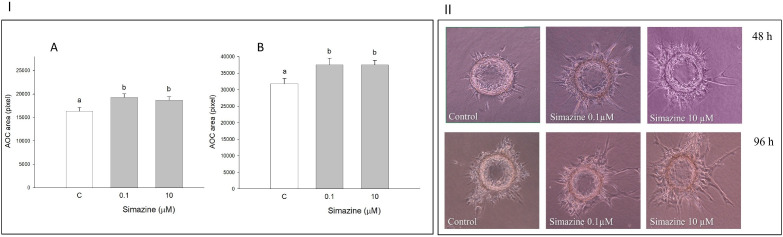
Panel I: Effect of the 48 (A) or 96 h (B) treatment with simazine (0.1 or 10 µM) on area covered by AOC in fibrin gel. Data represent the mean ± SEM of six replicates/treatment repeated in five different experiments. Different letters indicate a significant difference (P < 0.01) among treatments as calculated by ANOVA and Scheffè’ F test. Panel II: Phase contrast micrographs showing AOC growth in fibrin gel matrix after 48 or 96 h treatment with simazine (0.1 or 10 µM).

AOC incubated with simazine showed a higher growth rate as compared to controls (P < 0.01), both after 48 h and 96 h of incubation. The two different simazine concentrations displayed similar effects ( Figure 4 ; Panel I a and b; Figure 4 Panel II).

## Discussion

The chlorotriazines (atrazine, simazine, propazine and terbuthylazine) have been widely used as selective herbicides in agricultural crops or as total herbicides in the weed control in railways and roads. Atrazine in particular, is one of the most widely applied herbicides in the United States and it represents the most common water contaminant, having been found in nearly 70% of all surface and fresh ground water in the United States ( Bexfield, 2008 ). Due to their endocrine disruptor effects and their ubiquitous water contamination, atrazine and simazine use in agriculture has been prohibited in the European Union since 2004. As a consequence of their use in a massive scale, however, in Europe these compounds are still detected in groundwater ( Sassine *et al*., 2016 ; ISPRA Report 2016) and they remain the most ubiquitous pesticides in aquifers ( Loos *et al*., 2010 ). Their persistence in the environment for more than 10 years after their ban is a matter of concern for potential adverse health effects in mammalian and aquatic species. *In vitro* and *in vivo* studies have documented potential detrimental health effects for atrazine, among which the disruption of the neuroendocrine control of reproductive development and function in many animal species ( Cooper *et al*., 2000 ; Basini *et al*., 2012 ; Forgacs *et al*., 2013 ), the induction of mammary gland tumors in the rat ( Cooper *et al*., 2007 ) and the promotion of prostate cancer cell growth ( Hu *et al*., 2016 ).

With regard to simazine toxicity by Ren *et al*. (2013) has documented a possible impairment of immune function of mice orally exposed to simazine, while Stara *et al*. (2012) demonstrated that chronic exposure to this substance can induce changes redox status in common carp.

Starting from these experimental observations, showing that simazine can impair several different biological mechanisms, here we document that this compound, even at low doses, impairs swine granulosa cell function, possibly resulting in reproductive dysfunction. Rich *et al*. (2012) reported similar effects on human breast cancer cell lines, hypothesizing an association between simazine stimulatory action on cell growth and the presence of estrogen receptor.

Following a 24 h exposure of human granulosa cell-derived KGN cells to a wide range of simazine concentrations, Park *et al*. (2014) reported a biphasic response for cell viability and proliferation; in particular, low concentrations induced a dose-dependent decrease while higher dosages, in the range of those used in the present study, displayed no effect or stimulatory effects.

Extensive literature has been published about atrazine disrupting effects on steroidogenesis in ovarian granulosa and Leydig cells ( Basini *et al*., 2012 ; Fa *et al*., 2013 ; Pogrmic-Majkic *et al*., 2016 ); in particular, an atrazine-induced increase of progesterone secretion has been reported in different species ( Tinfo *et al*., 2011 ; Basini *et al*., 2012 ), raising concerns about a possible impairment of reproductive efficiency by atrazine even at low doses. On the contrary, information regarding simazine is scarce and its possible impact on reproductive activity is still far to be elucidated. Our data show that simazine increases progesterone production, while it does not modulate estradiol 17beta levels in cultured granulosa cells. Similar results have been documented by Forgacs *et al*. (2013) , who observed a simazine stimulatory effect on progesterone levels in murine Leydig cells, possibly due to changes in steroidogenic gene expression.

As regards to simazine possible modulatory action on estradiol 17beta production, a simazine-induced increase of aromatase has been documented in human adrenocortical carcinoma cell lines ( Fan *et al*. 2007 ; Sanderson *et al*., 2001 ). According to our present data, we cannot confirm the disrupting effects of simazine at micromolar range on estradiol production by swine granulosa cells, and thus its possible modulatory action should be further tested at higher dosages in the same *in vitro* model.

In previous works ( Basini *et al*., 2012 ) we have documented that atrazine, at dosages similar to those considered in the present work, can interfere with the angiogenic process within the ovarian follicles, in particular affecting the production of the main angiogenesis signaling molecules VEGF and NO.

Conversely to the observations of Park *et al*. (2016) about simazine suppressive effect on NO production in human granulosa cells, in our experiment this molecule appeared ineffective in modulating NO levels, while significantly enhanced AOC growth in the *in vitro* angiogenesis bioassay. The present results lead us to hypothesize that also simazine can be possibly qualified as a pro-angiogenic molecule, as already suggested for different endocrine disruptors ( Grasselli *et al*., 2010 ; Durando *et al*., 2011 ; Basini *et al*., 2012 ). Further studies are needed to better investigate about its potential modulatory effect on VEGF production, thus confirming this hypothesis.
